# Approaches for Studying Context Specificity of Translation Inhibitor Action

**DOI:** 10.3390/ijms27146365

**Published:** 2026-07-17

**Authors:** Ekaterina S. Komarova, Arina A. Nikandrova, Olga A. Dontsova, Petr V. Sergiev

**Affiliations:** 1 A.N. Belozersky Institute of Physico-Chemical Biology, Lomonosov Moscow State University, 119991 Moscow, Russia; arinanikandrova@mail.ru (A.A.N.); olga.a.dontsova@gmail.com (O.A.D.); petya@belozersky.msu.ru (P.V.S.); 2 Department of Chemistry, Lomonosov Moscow State University, 119991 Moscow, Russia; 3 Center for Bio- and Medical Technologies, 121205 Moscow, Russia; 4 Department of Biology, Lomonosov Moscow State University, 119234 Moscow, Russia; 5 Shemyakin-Ovchinnikov Institute of Bioorganic Chemistry, Russian Academy of Sciences, 117997 Moscow, Russia

**Keywords:** protein synthesis, inhibitor, context specificity, NGS, methodology, ribosome stalling, translation pausing

## Abstract

The sequence of messenger RNA (mRNA) not only determines the protein sequence synthesized by a ribosome but also defines the efficiency of this process. Many antibiotics lethal to bacteria inhibit various stages of translation by targeting ribosomal functional centers. Some antibiotics exhibit specificity not only for particular stages of the ribosomal working cycle but also for specific patterns within mRNA sequences. This review covers a broad range of approaches—including *in vivo* and *in vitro* methods, low- and high-throughput techniques such as reporter constructs, characterization of inhibitors of protein synthesis (ChIPS), toeprinting, cryogenic electron microscopy (cryo-EM), protein labeling, and those integrated with next-generation sequencing (NGS) like ribosome profiling with following NGS (Ribo-seq), inverse toeprinting coupled with NGS (iTP-seq), high-throughput toeprinting and NGS (Toe-seq), and ribosome display—used to study the sequence specificity of translation inhibitors, a rapidly evolving field crucial to molecular biology. It presents various methodologies, discusses their applications, and provides a comparative analysis. The fundamental research value of this review lies in establishing standardized experimental selection guidelines for scientists investigating ribosome stalling mechanisms, thereby minimizing trial-and-error costs. Equally important is its applied relevance. The review highlights its translational value in aiding the screening and mechanistic analysis of sequence-specific small-molecule inhibitors. Moreover, understanding the mechanisms underlying protein biosynthesis inhibition and their dependence on particular mRNA sequences could enable the development of selective agents that precisely suppress the synthesis of certain polypeptides, such as proteins from pathogenic bacteria or cancer-associated proteins.

## 1. Introduction

Translation is a crucial process of protein biosynthesis carried out by the ribosome, a macromolecular ribonucleoprotein complex. This complex decodes the genetic information encoded in an mRNA sequence and translates it into the amino acid sequence of a protein [1,2,3]. Ribosomes interact with multiple ligands, including mRNA, transfer RNA (tRNA), and translation factors, to perform protein synthesis. It is well established that the mRNA sequence not only determines the protein sequence but also influences the efficiency of protein biosynthesis, which can vary by up to four orders of magnitude [4,5]. Each mRNA can be characterized by several properties that affect the translation rate and efficiency, such as nucleotide composition of a 5′-untranslated region (5′-UTR) [4,6,7], secondary structure elements [6,7,8,9,10,11,12], codon usage at the start of an open reading frame (ORF) [10,11,12,13,14,15,16,17], oligo- or polyproline stretches [18,19,20], and regions encoding stop peptides that interact with the ribosome nascent peptide exit tunnel (NPET) [21,22].

Translation is also a target of various antibiotics, which can inhibit cell growth or cause cell death by binding to ribosomal functional centers and interfering with protein biosynthesis at different stages [23,24]. Initially, such inhibitors were considered non-selective with respect to protein sequences [25]. However, the active application of both classical and modern methods is changing this paradigm [26,27,28,29,30,31,32]. Emerging evidence suggests that some antibiotics act in a sequence-specific manner, selectively blocking translation at particular motifs within nascent peptides [26,27,28,29,31,32,33,34,35,36,37,38].

Mankin’s group was the first to show that macrolides—clinically important antibiotics that block NPET—allow selective protein synthesis when bound to ribosomes, thereby reshaping the cellular proteome without globally halting translation [31]. In this context, the nascent peptide sequence determines its ability to bypass the drug-obstructed tunnel. While the synthesis of some polypeptides is arrested early during translation, others may be halted at later elongation stages, and some proteins can be produced despite the presence of an antibiotic. Thus, the binding of small molecules within the ribosomal exit tunnel may render it selective to particular protein chains. These effects were primarily detected using protein isotope labeling, two-dimensional (2D) gel electrophoresis, subsequent mass spectrometry protein identification, and classic toeprinting analysis in a bacterial *in vitro* system. The macrolides studied include erythromycin (ERY), the second-generation azithromycin (AZI), and newer-generation ketolides such as telithromycin (TEL) and solithromycin (SOL) [31].

Shortly thereafter, Sergey Dmitriev and co-workers demonstrated, using a eukaryotic cell-free translation system and the toeprinting technique, that the plant alkaloid harringtonine (HT) and its close derivative, homoharringtonine, specifically halt elongating ribosomes at Lys, Arg, and Tyr codons positioned in the P-site [32]. Molecular modeling further suggested that the amino acid specificity of HT and its analog is dictated by the distinct architecture of the peptidyl transferase center (PTC), which is occupied by peptidyl-tRNAs bearing Lys, Arg, or Tyr residues at the C-terminus of the nascent peptide [32]. The following year, in 2014, Mankin’s group applied ribosome profiling to deduce the rules governing macrolide sequence specificity [39].

Over time, the number of antibiotics exhibiting context-specific inhibition has increased, as identified through both low- and high-throughput methods [33,34,35,36,37,38,39,40,41,42,43,44,45,46,47,48,49,50,51,52,53,54,55,56,57,58,59]. This list was expanded to include the aminocyclopentitol pactamycin (PAC) [26,34], the broad-spectrum drug chloramphenicol (CHL) and the synthetic oxazolidinone linezolid (LZD) [36,45], as well as the natural ketolides methymycin (MTM) and pirkomycin (PKM) [37]. Several additional PTC inhibitors—anisomycin, blasticidin S, sparsomycin, trichothecene antibiotic T-2 toxin, diacetoxyscirpenol, and others—have demonstrated amino acid specificity by producing distinct ribosome stalling patterns in eukaryotic *in vitro* translation systems [38,47].

A small-molecule inhibitor, PF-06446846 (PF846), which binds to the human ribosome, was shown to abolish the production of specific proteins [40,44]. Furthermore, the macrolide oleandomycin (Ole) [43], the aminoglycoside kasugamycin (KSG) [26,41,49], and the aromatic polyketide tetracenomycin X (TcmX) [46,53,54] have been added to this list. Notably, telithromycin’s context-specific action on eukaryotic ribosomes harboring a single ribosomal RNA (rRNA) mutation was also demonstrated [48].

In the following five years, additional antibiotics exhibiting mRNA sequence specificity have been described, including the synthetic oxazolidinone radezolid (RZD) [50], the oligosaccharide orthosomycin evernimicin (EVN) [51], the aromatic anthracenopyranone thermorubin (THR) [52], and etamycin A (EtaA) from the streptogramin B group [54]. Recently, mitoribosome profiling revealed that CHL and LZD also induce mitoribosome stalling in a context-specific manner [55]. Commonly used antibiotics to control ribosome halting at the start codon include retapamulin (RET) [52], tetracycline (TET), and thiostrepton (THS) [54], while borrelidin (BOR) [54] is used for stalling at the threonine codon.

Over the past year, new insights have been reported regarding the sequence-specific mechanisms of bottromycin A2 (BotA2) [56,57], a ribosomally synthesized and post-translationally modified peptide (RiPP); manikomycin (MKM) [58], a natural depsipeptide; and a benzoxaborole derivative of azithromycin (Azi-BB) [59].

These data indicate that the ribosome nascent peptide exit tunnel is a dynamic environment where the synthesized peptide, ribosome, and inhibitor interact in a complex, sequence-dependent manner [54]. Understanding these intricate interactions could reveal new avenues for altering antibiotic selectivity and overcoming antibiotic resistance. The sequence specificity of antibiotic action may contribute to enhancing antimicrobial properties but also to broadening fundamental knowledge of the ribosomal response to environmental cues [42].

While the influence of mRNA sequence on translation efficiency is well studied [4,6,7,10,30,60,61,62,63,64], the investigation of mRNA sequence specificity of translation inhibitors is an actively developing field [41]. Complete cessation of protein biosynthesis is likely to lead to a bacteriostatic effect, whereas selective inhibition of the translation of certain proteins can disrupt cellular proteome balance, resulting in a loss of viability [31].

Another promising possibility is the creation of selective inhibitors targeting specific mRNAs [40] and, thus, suppressing the synthesis of a limited set of proteins. Although the design of such molecules remains infrequent, the need for them is urgent. To advance this field, it is essential to be familiar with the various approaches available for studying the specificity of antibiotic action.

Over the decades, various methods have been developed to decipher antibiotic context specificity [65,66,67,68,69,70,71,72,73,74,75,76,77,78,79,80,81,82,83,84,85,86,87]. These approaches can be categorized based on criteria such as *in vivo* or *in vitro* application, experimental complexity, and low- or high-throughput levels.

Among low-throughput *in vitro* assays, toeprinting [65,66,67] is regarded as the gold standard. It is used both to estimate translation efficiency and to map drug-induced ribosome stalling patterns on mRNA, monitored via primer extension [31,32,33,34,35,36,37,38,43,46,49,50,51,52,53,54,56,57,58,59].

Cryo-EM deserves special mention, as it allows the visualization of structural interactions between the ribosome, tRNAs, translation factors, mRNA, and antibiotics during translation *in vitro*, often at resolutions surpassing those of crystallography [42,46,48,50,53,54,55,68,69,70,71,72,73].

Several approaches have also been adopted to test translation inhibition *in vivo* at the level of single mRNA templates to detect or confirm ribosome stalling on specific motifs [28,29,43,53,68,74,75,76,77]. Most of these methods rely on reporter constructs [28,43,53,68,74,76,77].

Another two-pronged tool for describing protein synthesis inhibitors is ChIPS [34]. This technique combines the *in vivo* use of engineered antibiotic-hypersensitive *Escherichia coli* (*E. coli*) strains containing a single rRNA operon for rapid isolation of resistant mutants with mutations at the antibiotic action site, followed by *in vitro* toeprinting to elucidate the antibiotic’s mode of action.

Among large-scale *in vivo* methods, protein labeling is notable for monitoring global patterns of synthesized proteins in living cells, followed by protein isolation and mass spectrometry to identify peptide sequences [31,35,37,48,49,51,78,79]. This approach can also be applied *in vitro*.

The application of breakthrough next-generation sequencing has significantly contributed to the development of new high-throughput assays [4,30,80] for profiling the cellular transcriptome, identifying efficiently translated mRNAs, and detecting ribosome stalling on natural or synthetic mRNA libraries. Among the most useful *in vivo* approaches, Ribo-seq stands out. This technique uses sequencing of mRNA regions protected by translating ribosomes in living cells [30,36,55] and has proven highly effective for studying gene expression at both transcriptional and translational levels [81,82,83].

A related method, iTP-seq, relies on ribosome protection of randomized mRNA fragments from RNase R cleavage *in vitro* [43,53,68]. Another *in vitro* assay for large-scale selection of peptides or proteins—resulting in highly effective binders against virtually any antigen—is ribosome display [84]. Adapted for use in both bacteria and eukaryotes, this approach has also been modified to determine the context specificity of a small-molecule inhibitor of human translation [44].

Recently, our laboratory, in collaboration with Kabilov’s group, developed an *in vitro* technology combining high-throughput toeprinting with NGS, called Toe-seq [54,56,59]. This method enables simultaneous monitoring of *in vitro* translation of an mRNA library encompassing 10^4^–10^5^ variants under normal conditions or in the presence of various antibiotics.

Another large-scale technique, STALL-seq (Selection of Translational Arrest sequences from Large Library sequencing) [85], identifies ribosome stalling sites across randomized sequences and is applicable to both bacterial and eukaryotic systems.

Two additional assays include low-throughput *in vivo* and *in vitro* nascent chain profiling (iNP) [86] and high-throughput *in vivo* genetic selection for sequences inducing ribosome stalling [87]. These methods reveal sequences containing ribosome arrest motifs under normal conditions and can potentially be adapted to detect ribosome halting triggered by translation inhibitors.

This review aims to provide a detailed overview of diverse approaches used to identify ribosome stalling motifs and verify the sequence specificity of various translation inhibitors. It systematically classifies all *in vivo* and *in vitro*, low- and high-throughput experimental assays, and conducts a horizontal comparative analysis of each technique, highlighting their advantages, limitations, compatible biological systems, and representatives of different inhibitor categories for which they were used. Furthermore, it proposes scenario-based experimental selection strategies and combined validation workflows that integrate low- and high-throughput platforms to guide future mechanistic research and drug development.

## 2. Variety of Approaches for Studying the Context Specificity of Translation Inhibitor Action

### 2.1. Single mRNA Methods for Studying Context Specificity of Translation Inhibitors

#### 2.1.1. *In Vivo* Methods: Reporter Constructs and Strains

Several natural sensors of translation inhibitors, such as macrolides, lincosamides, streptogramin B group (MLS) antibiotics, and chloramphenicol, which regulate resistance gene expression, rely on arresting ribosome movement along specific mRNA sequences [28,43,53,68,88,89,90,91,92]. Attenuation systems regulate the expression of various genes, including the *erm* family responsible for MLS resistance [89]. Erythromycin resistance methyltransferases (Erms) modify the residue A2058 of 23S rRNA (*E. coli* numbering used throughout) [68]. These enzymes are generally encoded within operons containing both a leader peptide gene and a resistance gene [28,88,89,91,92]. Interestingly, some macrolides and their derivatives do not induce *erm* expression [90]. A similar activation mechanism has been described for genes conferring resistance to CHL [93]. Our laboratory applied the same principle to develop an attenuation-based dual-fluorescent protein reporter system for general screening of translation inhibitors, as well as SOS-response inducers (Table 1) [46,74,76,77,94,95,96,97,98].

**Figure 1:**
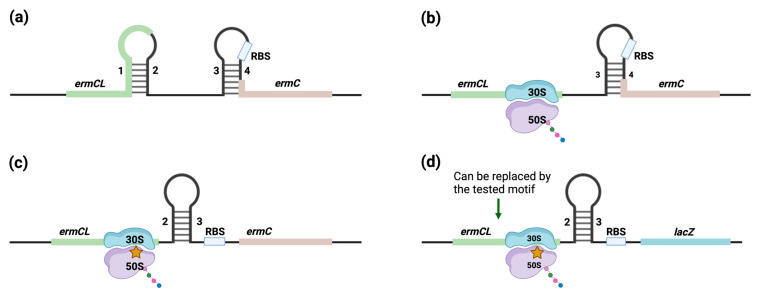
Regulation of *ermC* expression. (**a**) Secondary structure of the *ermC* mRNA in the absence of a macrolide antibiotic. (**b**) Translation of the *ermCL* leader peptide maintains the inhibitory mRNA structure and prevents *ermC* expression. (**c**) Macrolide-induced ribosome stalling (macrolide marked by a star) remodels the mRNA structure, exposing the RBS and enabling *ermC* translation. (**d**) In the reporter-based sensor construct, *ermC* is replaced by *lacZ*; the *ermCL* region can be replaced by a sequence with a ribosome stalling motif. The growing peptide is represented as a chain of beads. Created in BioRender. Sergiev, P. (2026) https://BioRender.com/epc23r3 (accessed on 11 June 2026).

The most studied attenuation system induced by antibiotics is the *ermCL-ermC* operon (Figure 1a) from *Staphylococcus aureus* [90]. In the absence of an inhibitor, Erm(C)L is efficiently translated (Figure 1b), while the ribosome binding site (RBS) of *erm(C)* is masked by the mRNA secondary structure, rendering it inaccessible [28,101]. Antibiotic-induced arrest of Erm(C)L translation reconfigures the mRNA secondary structure, facilitating Erm(C) synthesis (Figure 1c). This natural mechanism inspired the creation of an MLS antibiotic sensor by replacing the methyltransferase gene with a *β*-galactosidase (*lacZ*) reporter gene (Figure 1d) [28,53,68,92,102].

Such reporters can be used to test whether an antibiotic stalls the ribosome at a particular sequence, which should be incorporated into the translated leader region [43,53]. For example, replacement of the MGIFSIFVIS motif in ErmCL with MAAAPQKC converted it into a sensor for TcmX [53].

Reporter assays are widely used in both bacteria and eukaryotes. However, they have inherent limitations: they are only suitable for validating known arrest motifs and cannot be used for high-throughput screening of uncharacterized novel sequences. Additionally, false-positive signals may arise due to interference from mRNA secondary structures.

A combined approach for characterizing protein biosynthesis inhibitors, termed ChIPS, has been designed [34]. It involves an *in vivo* stage using engineered antibiotic-hypersensitive *E. coli* strains containing a single rRNA operon, enabling rapid isolation of resistant mutants carrying rRNA mutations at the inhibitor’s action site. This is followed by an *in vitro* stage that monitors primer extension patterns of drug-induced ribosome arrest on mRNA, elucidating the antibiotic’s mode of action. These techniques have been successfully validated with a bacterial strain exhibiting translation inhibition activity and facilitated the rapid identification of CHL’s presence in culture extracts [34].

The use of reporter constructs and strains enables testing the effect of a specific inhibitor on the translation of a particular mRNA sequence in living cells; however, it is a low-throughput approach that requires a lengthy and costly process to verify even a few mRNA sequences of interest.

#### 2.1.2. *In Vitro* Methods: Toeprinting, Cryo-EM

**Figure 2:**
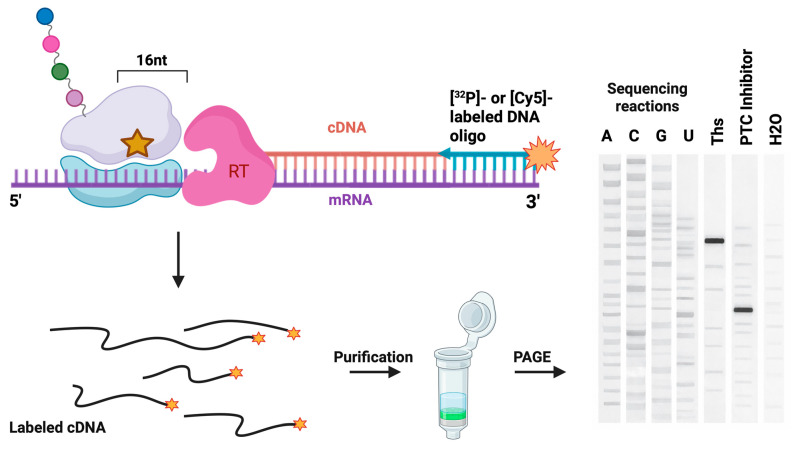
Toeprinting assay for detection of inhibitor-induced ribosome stalling. Ribosome stalling or pausing is detected by primer extensions using labeled primers (labels are indicated by polygons at the primer ends, inhibitor—by a star). The growing peptide is represented as a chain of beads. Full-length or truncated cDNA products (toeprints, indicated as bands on the gel) are resolved by denaturing polyacrylamide gel electrophoresis (PAGE) along with the mRNA sequencing reactions to map ribosome positions with single-nucleotide resolution. Black major bands on the gel mark the ribosome stalling positions. Created with BioRender. Sergiev, P. (2026) https://BioRender.com/j15okwl (accessed on 11 June 2026).

Among the widely applied *in vitro* methods for testing ribosome arrest on specific mRNA sequences caused by translation inhibitors, the toeprinting assay is considered the gold standard (Figure 2, Table 1) [65,66,67]. Toeprinting is based on reverse transcription (RT) of mRNA from a primer complementary to a region downstream of the ribosome position, typically in the 3′-UTR. This primer can be labeled fluorescently or radioactively ([^32^P]) to detect the resulting complementary DNA (cDNA). Reverse transcriptase synthesizes cDNA from the mRNA template until it encounters a translating ribosome or, if no ribosome is bound, until it reaches the 5′-end of the mRNA [64]. Because the ribosome covers a substantial region of mRNA, RT typically terminates 16 nucleotides (nt) downstream from the first nucleotide of the codon in the ribosome’s P-site [52,103,104,105]. Occasionally, due to the ribosome’s dynamic structure, this distance can be 17 nt, but codon resolution is still maintained according to mRNA sequencing data. Comparison of band intensities corresponding to the full-length cDNA and shorter products representing translation intermediates can be used to deduce translation efficiency. More importantly, the distribution of cDNA lengths pinpoints ribosome stalling or pausing positions along the mRNA coding sequence [65,66,67]. Ribosome stalling induced by an antibiotic at a specific mRNA sequence appears as an accumulation of a distinct cDNA product [33,34].

Toeprinting is a low-throughput approach for determining the sequence specificity of translation inhibitors since it examines one mRNA sequence and one inhibitor effect at a time [35,46,52,53,54]. Numerous antibiotics targeting ribosomes—including MLS, BOR, CHL, oxazolidinones, PAC, KSG, EVN, TcmX, TET, THR, THS, RET, EtaA, BotA2, MKM, and others—have primarily been tested using toeprinting with *h-ns*, *osmC*, *ermBL*, *ermCL*, *yrbA*, and *rst1-3* mRNA templates, which contain codons for nearly all proteinogenic amino acids [31,32,33,34,35,36,37,38,43,46,49,50,51,52,53,54,56,57,58,59]. This technique has also been adapted for eukaryotic cell-free translation systems coupled with RT and successfully applied to detect ribosome halting caused by the NPET inhibitor PF846 [40,44], as well as PTC inhibitors such as HT, T-2 toxin, anisomycin, blasticidin S, sparsomycin, diacetoxyscirpenol, and others [32,38].

In recent years, cryo-electron microscopy has emerged as a powerful tool for visualizing the three-dimensional (3D) structures of biological molecules and complexes, achieving near-atomic resolution [106]. The electron beam passes through the sample, producing a series of 2D projections, which are then reconstructed into a 3D model using advanced computational algorithms [99,107].

Cryo-EM has found widespread application in detailed studies of the ribosome, revealing its spatial interactions with tRNAs, translation factors, mRNA, and various ligands. Significant advances in the resolution of crystallographic and cryo-EM reconstructions have been crucial for identifying antibiotic interactions with functional ribosome complexes [41,42,52,69,103,104,108,109,110,111,112]. This technique provides a structural explanation for the sequence specificity of various ribosomal inhibitors.

Cryo-EM studies confirmed macrolide-dependent ribosome stalling at various macrolide-arrest motifs (MAMs) [42], primarily found in regulatory upstream open reading frames (uORFs) of macrolide resistance genes. ErmBL-dependent translational arrest occurred with both macrolides (ERY, Ole) and ketolides (SOL, TEL), contrasting with other leader peptides such as ErmCL, where stalling was specific to macrolides [28,71,72]. Stalled ribosome complexes (SRCs) with ErmDL and ERY or TEL revealed ribosome arrest during synthesis of an Arg-Leu-Arg motif, with average resolutions of 2.9 Å and 3.1 Å, respectively [68]. ERY showed less stringent sequence specificity compared to TEL. Cryo-EM structural analysis also demonstrated TEL’s ability to bind within the NPET of an engineered eukaryotic ribosome (yeast G2400A mutant) at an average resolution of 3.1 Å [48]. Complementary studies using crystallography and cryo-EM provided insights into the context specificity of macrolide inhibition during bacterial translation, even at lower resolutions [69,73,112].

Several high-resolution cryo-EM studies have clarified the structural basis of context-specific action of ribosome inhibitors such as RZD and LZD in complexes with the MFKAF stalling peptide [50]; TcmX with mRNA encoding the MAAAPQKCAAA peptide, where ribosome halting occurs at the Gln-Lys (QK) motif [53]; and EtaA with mRNA containing the MVVK arresting motif and ribosome resolved at 2.2 Å [54]. Other examples include CHL blocking the 70S ribosome bound to the poly(A)KAAD nascent peptide [70], and both CHL and LZD stalling mitochondrial ribosomes in complexes with peptide sequences MMYALF and MMYYLF [55].

For a long time, detailed information about inhibitor-induced ribosome stalling sites in specific mRNA sequences was mainly provided by *in vitro* approaches such as toeprinting and cryo-EM, which have been adapted to both bacterial and eukaryotic translation systems. However, these low-throughput methods do not allow for the analysis of an entire mRNA library simultaneously to identify patterns of antibiotic stalling sites. Moreover, toeprinting requires careful sequencing to precisely map the exact ribosome stalling positions, while cryo-EM involves the laborious process of obtaining highly purified drug-bound ribosome complexes, collecting multiple images, and using deep learning software (e.g., built directly into the Relion v.4.0 [53]) to enhance resolution and overcome low signal-to-noise ratios. Recently, these approaches, primarily using single mRNA types, have been employed to verify and refine findings on the context-specific action of translation inhibitors obtained by high-throughput methods, which will be discussed below [35,36,39,42,43,48,49,51,53,54,68].

### 2.2. High-Throughput Methods for Studying Context Specificity of Translation Inhibitor Action

#### 2.2.1. *In Vivo* Methods: Protein Labeling, Ribo-Seq

**Figure 3:**
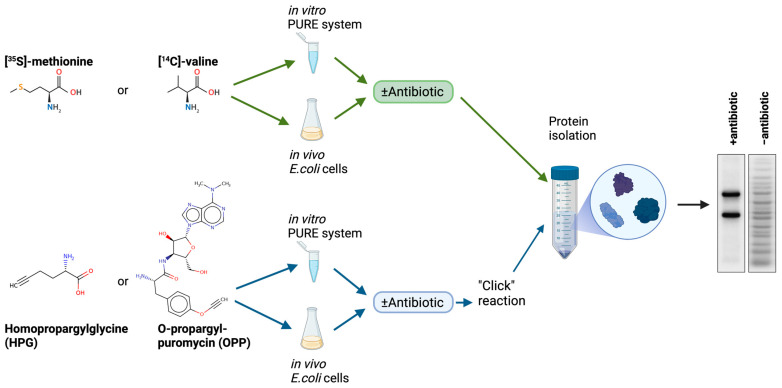
Workflow for metabolic labeling-based analysis of protein synthesis under control and antibiotic-treated conditions. Newly synthesized proteins, *in vivo* or *in vitro*, are labeled using radioactive amino acids or non-radioactive analogs. Extracted proteins (indicated as bands on the gel) are then resolved by PAGE either directly or after an additional “click” reaction for non-radioactive labeling. Comparing control and antibiotic-treated samples reveals a strong global inhibition of translation, accompanied by continued synthesis of a distinct subset of proteins that remain efficiently translated despite the presence of ribosome-targeting antibiotics. Created in BioRender. Sergiev, P. (2026) https://BioRender.com/jgrw76d (accessed on 11 June 2026).

To monitor the global pattern of synthesized proteins in living cells, various labels can be used, categorized as radioactive or non-radioactive. The first category typically involves isotopes such as [^35^S] or [^14^C], which can replace sulfur or carbon atoms, respectively, in amino acids. For example, [^35^S]-methionine ([^35^S]-Met) and [^14^C]-valine ([^14^C]-Val) are commonly incorporated into proteins during their active synthesis *in vivo* or *in vitro* [41,42,48]. The second category includes molecules like homopropargylglycine (HPG) and O-propargyl-puromycin (OPP), which can be incorporated into newly synthesized peptide chains and visualized via a chemoselective copper(I)-catalyzed ligation (“click” reaction) between their alkyne group and an azide-containing fluorescent dye [79,113,114]. HPG is a methionine analog [79,114] incorporated into protein chains during natural protein synthesis, while OPP is a puromycin analog that forms covalent conjugates during premature release of nascent protein chains [113]. Bulk newly synthesized proteins from living cells can be extracted and analyzed by one- or two-dimensional polyacrylamide gel electrophoresis. This technique, using radioactive labels [^35^S]-Met and [^14^C]-Val, has been applied to analyze *de novo* synthesized proteins in normal *E. coli* cells and after treatment with various antibiotics (Figure 3, Table 1), including the macrolide ERY and the ketolides TEL and PKM [35,78]. Remarkably, cells exposed to high drug concentrations continue to selectively produce a significant subset of proteins at levels comparable to those in untreated cells [31,37]. These proteins can be isolated and identified by mass spectrometry to determine their sequences [79].

Moreover, this method has been used to analyze proteins translated *in vitro* with the isotope label [^35^S]-Met. In a modified version, it was combined with SOL labeled with [^14^C] to verify the inhibition of protein synthesis by this extended macrolide in mutant yeast cells, which act upon ribosomes [48,100].

**Figure 4:**
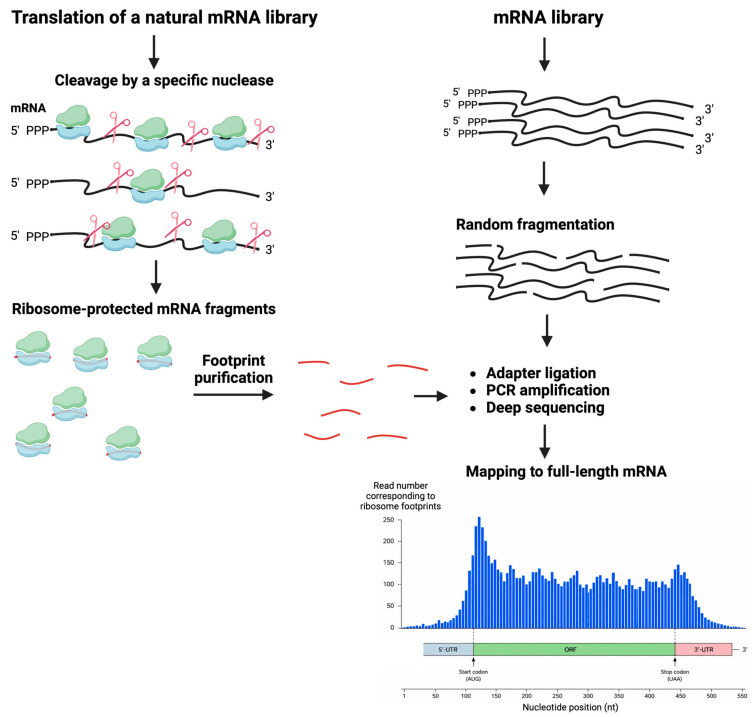
Ribo-seq workflow for genome-wide translation analysis. Actively translating ribosomes stall and protect fragments of mRNA. Following specific nuclease cleavage (indicated by scissors), ribosome-protected fragments (shown by red lines) are isolated and prepared for NGS. Sequencing of a randomly fragmented total mRNA library provides a reference sequence. Mapping RPFs to the reference transcriptome reveals ribosome occupancy along mRNAs and translation efficiency across the transcriptome. Created in BioRender. Sergiev, P. (2026) https://BioRender.com/0te4595 (accessed on 11 June 2026).

The rapid advancement of next-generation sequencing technologies and the accumulation of expertise in translation systems have led to the development of the *in vivo* ribosome profiling method (Figure 4, Table 1) [30,115,116,117]. Ribo-seq reveals ribosome distribution along translated mRNAs in living cells: peaks of ribosome density accumulate at codons where protein biosynthesis slows down, while codons traversed more quickly have fewer associated ribosomes [42,116]. The technique is based on isolating ribosome-protected mRNA fragments (RPFs or footprints), followed by deep sequencing and mapping to the reference genome sequence [30].

The multi-step procedure begins with polysome extraction from rapidly frozen cells, followed by digestion with ribonuclease (RNase) I [30,115] or micrococcal nuclease (MNase) [35,116] to generate monosomes with ribosome footprints. Ribosomes bound to footprints approximately 28–30 nt long are isolated by sucrose density gradient ultracentrifugation. The mRNA fragments are then purified through size selection—typically 25 to 45 nt—using PAGE, followed by further purification [30,115,117]. In parallel, total RNA is analyzed by NGS to normalize ribosome footprints against RNA abundance.

Generating sequencing libraries from footprint fragments poses challenges similar to those encountered with endogenous small RNAs such as microRNAs, requiring extensive optimization. A protocol involves 3′-end dephosphorylation of mRNA fragments generated by nuclease treatment or alkaline hydrolysis to create a 3′-hydroxyl group [115]. Because different RNA ligases exhibit sequence preferences when attaching linker sequences [118], polyadenylated sequence (poly(A)) polymerase is employed to produce more uniform libraries with polyadenylated 3′-ends [30].

Reverse transcription is then performed on both libraries using a custom oligonucleotide containing an anchored repeating deoxythymidine residues (oligo-d(T)) primer at the 3′-end, a flexible spacer, and a reverse complement linker sequence at the 5′-end. The first-strand cDNA is circularized using single-stranded (ss) DNA ligase, followed by polymerase chain reaction (PCR) amplification with primers complementary to linker sequences flanking the target RNA fragment [115]. PCR products are purified via non-denaturing PAGE. After purification, barcodes are added, and samples are prepared for NGS [83,117]. The resulting ribosome-protected footprint sequences are mapped to the genome, and their coverage is analyzed [30].

This breakthrough approach has proved effective for simultaneously studying gene expression at both transcriptional and translational levels, including responses to various influences [81,82,83]. It allows the determination of average ribosome occupancy at every codon within a gene, providing a comprehensive view of the translational landscape in cells [41,42]. Ribo-seq offers single-nucleotide resolution of ribosome positions on mRNA, enabling the detection of translation in overlapping ORFs, regions outside annotated ORFs, and deciphering of stop codon usage [64]. This method has identified multiple regions previously considered non-coding as translatable. Furthermore, Ribo-seq enables the evaluation of how various conditions and factors—such as protein modifications, environmental changes, and antibiotics—affect mRNA translation in cells [119,120,121,122,123,124].

Comparing codon occupancy between antibiotic-treated and untreated cells reveals mRNA sites where drugs most strongly inhibit translation: these codons show increased occupancy in treated cells, while codons where the ribosome evades inhibition show decreased occupancy [41].

Multiple Ribo-seq experiments have been pivotal in determining the context specificity of translation inhibition. Genome-wide ribosome profiling revealed that macrolides such as AZI [35], ERY, and TEL [39] preferentially inhibit translation at the consensus Arg/Lys-X-Arg/Lys (+X+) motif, consistent with known MAMs [42]. Other motifs in strong ERY-induced arrest sites include XDK and XP [39]. Later, context-specific translation inhibition by CHL and LZD was identified: CHL-induced stalling preferentially occurs at Ala codons, and to a lesser extent when Ser or Thr codons are located in the E-site, with LZD showing an even stronger preference for Ala at the same position [36].

Ribosome profiling has also been used to assess sequence-specific inhibition by a broader range of antibiotics, including KSG [49], EVN [51], and RET [83]. The specificity of the latter in blocking translation immediately after initiation is exploited in the Ribo-RET method to map bacterial start codons [83,123]. TEL binding within the NPET of an engineered yeast cytoplasmic ribosome harboring the G2400A mutation in 25S rRNA was studied [48], and the effects of CHL and LZD on mitochondrial ribosomes were examined via adapted mitoribosome profiling [55].

The widespread use of ribosome profiling has revealed artifacts and challenges in experimental methodology and data analysis [125,126,127]. Although highly valuable, the Ribo-seq technique often exhibits uneven coverage along mRNAs that cannot be fully explained by actual ribosome occupancy. To address this data heterogeneity, various normalization methods have been developed. Significant variability in read distribution across mRNAs and determinants of ribosome footprint occurrence were observed in thirty publicly available Ribo-seq datasets, raising concerns about the method’s accuracy in identifying local ribosome density without rigorous quality control [125]. This highlights an incomplete understanding of how protocol parameters impact ribosome footprint density, likely due to sequence bias introduced by nucleases [30,115,116,117,128] and during later stages of library preparation for sequencing [126]. Importantly, the sequence specificity of antibiotics used to freeze translation [36,41,48] must be considered when aiming to accurately decipher the natural translation landscape of a cell.

The described *in vivo* large-scale methods provide a comprehensive view of protein translation in living bacterial and eukaryotic cells. However, protein labeling cannot identify antibiotic-induced ribosome stalling sites; it only detects the remaining synthesized proteins under drug treatment. In the case of Ribo-seq, multiple stages involved in preparing the footprint library for NGS introduce biases in its distribution across the transcriptome, and it may fail to reveal stalling patterns for diverse mRNAs if the inhibitor lacks strong sequence specificity. Additionally, false-positive ribosome stalling can occur due to factors other than the drug.

#### 2.2.2. *In Vitro* Methods: iTP-Seq, Toe-Seq, and Ribosome Display

**Figure 5:**
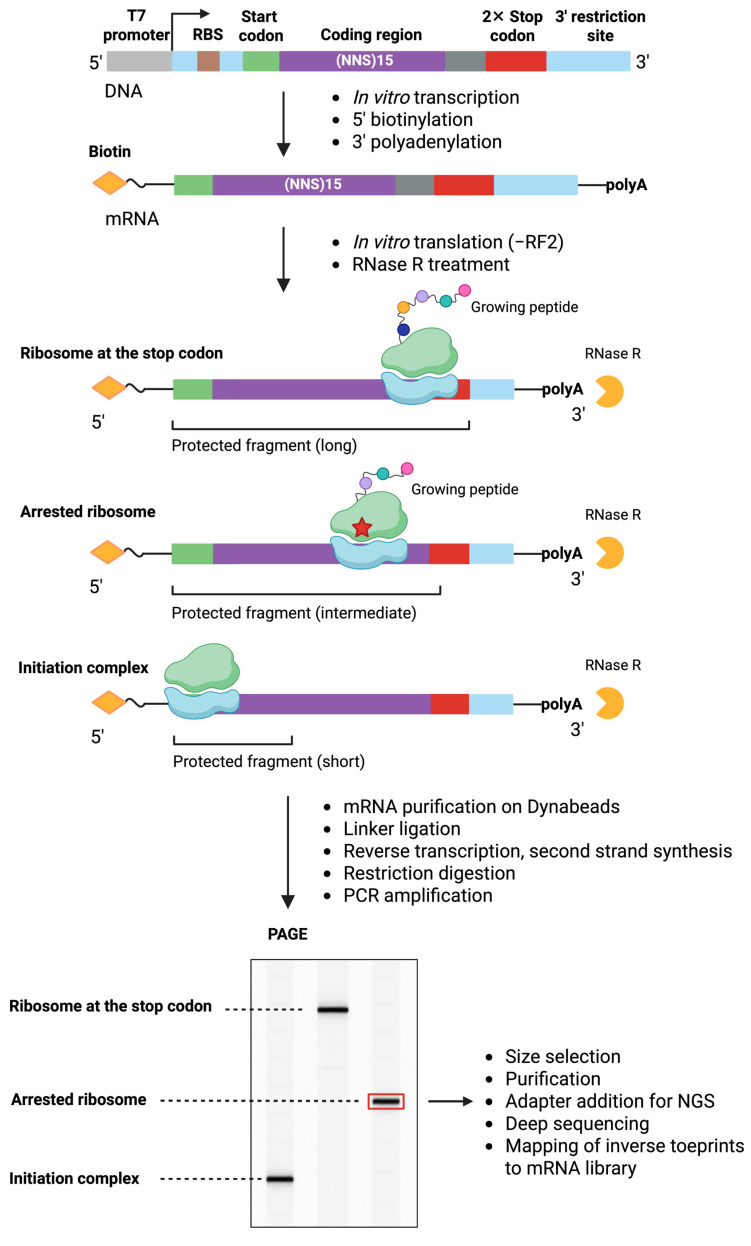
Workflow of iTP-seq for high-throughput analysis of antibiotic-induced ribosome stalling. A biotinylated and polyadenylated mRNA library with a randomized coding region is translated *in vitro*, followed by RNase R digestion to generate ribosome-protected fragments (inverse toeprints). The growing peptide is represented as a chain of beads, antibiotic–by a star. After affinity purification and linker ligation, inverse toeprints are reverse-transcribed, PCR-amplified, and size-selected by PAGE to isolate fragments corresponding to ribosome stalling within the randomized region (indicated as a major band on the gel within a red rectangle). Sequenced fragments are then mapped to the mRNA library, allowing identification of ribosome arrest motifs with codon-level resolution. Created in BioRender. Sergiev, P. (2026) https://BioRender.com/muwq0zn (accessed on 11 June 2026).

In ribosome profiling, mRNA library diversity is limited to the natural variants present in living cells, which, although diverse, may be biased due to evolutionary selection imposed on mRNA sequences. In contrast, synthetic libraries randomize most of the coding region, thereby unifying other mRNA fragments. To study the context specificity of translation inhibitor action *in vitro*, a growing variety of high-throughput techniques has emerged. One key method is inverse toeprinting combined with deep sequencing (Figure 5, Table 1) [43,129].

Unlike Ribo-seq, which captures short ribosome footprints, iTP-seq retains the entire coding region upstream of stalled ribosomes along with the 5′-UTR, enabling the study of random or targeted mRNA libraries and covering a broader sequence space. This method employs the highly processive exonuclease RNase R, which degrades mRNA transcripts from their 3′ poly(A) tail in the 3′ to 5′ direction until it reaches the leading ribosome. RNase R cleavage consistently occurs at +17 nt downstream of the ribosomal P-site, allowing precise codon-resolution mapping of ribosome stalling positions *in vitro* [43].

The synthetic DNA template libraries, (NNS)_15_ or (NNN)_15_, contain a T7 promoter, a 5′-UTR with an RBS, an ORF with a variable region, and a 3′-UTR. The ORF encodes 20-residue peptides starting with ATG codon, followed by a randomized region of 15 codons composed of either NNS (N = A/C/G/T; S = G/C) [43,68] or NNN (any nucleotide equally) [53] codons, plus four spacer codons upstream of two TGA stop codons. The (NNS)_15_ library avoids additional TGA or TAA stop codons within the randomized region. Following *in vitro* transcription by T7 RNA polymerase, the DNA library is converted into mRNA, then 5′-biotinylated and 3′-polyadenylated for purification on Dynabeads and efficient RNase R degradation, respectively [129].

The mRNA library is translated *in vitro* with release factors 1 and 3 (RF1, RF3) present, but release factor 2 (RF2) absent. Ribosomes stall if arrest motifs exist in the randomized region or continue to the stop codon downstream of the spacer codons. RF1 releases peptides at UAG stop codons inside the randomized region for the (NNS)_15_ library, or at UAG and UAA stop codons for the (NNN)_15_ library, while RF2 absence prevents release at UGA stop codons both within the randomized region and at the ORF end. RNase R degrades mRNA from the 3′-end up to stalled ribosomes, protecting the upstream coding region [43].

mRNAs are purified via streptavidin-bound Dynabeads using the 5′-biotin, which removes RNase R, ribosomes, and degraded fragments. A 5′-adenylated, 3′-blocked ssDNA linker is ligated to the mRNA 3′-end using truncated T4 RNA ligase 2 [43]. Reverse transcription is performed with a primer annealing to the linker, followed by second-strand synthesis using a complementary primer. The resulting double-stranded DNA (dsDNA) is treated with a restriction enzyme that cuts only the linker sequence, which is protected from RNase R degradation by ribosomes stalled at the UGA stop codon at the end of the ORF. This allows selective PCR amplification of dsDNA derived from mRNAs containing ribosome-stalling peptides within the randomized region [129].

PCR products are size selected by PAGE to isolate inverse toeprints corresponding to ribosome stalls within the randomized region, excluding initiation or termination complexes. Purified DNA fragments are then prepared for NGS by adapter ligation and sequenced [129].

Purified cDNA can undergo PCR amplification with specific primers for plasmid insertion and *in vivo* verification of stalling motifs, or can be subjected to additional rounds of inverse toeprinting by re-adding T7 promoter, 5′-UTR, start codon, and linker sequences. Given the large size of initial mRNA libraries, multiple rounds of selection enrich efficient ribosome arrest sequences [43].

iTP-seq has characterized stalling landscapes of free and drug-bound ribosomes with antibiotics such as ERY [43,68], Ole [43], TEL [68], and TcmX [53]. ERY showed predominant enrichment of the +X+ arrest motif and the general XPX motif, including subsets +XW and XPW [43]. TcmX-associated ribosomes exhibited a 1.7-fold increase in translation inhibition at the QK motif (Gln in the E-site, Lys in the P-site) among approximately 65,500 variants, with additional enrichment of PQKC stalling when Pro occupies the −2 position and Cys is in the A-site [53].

**Figure 6:**
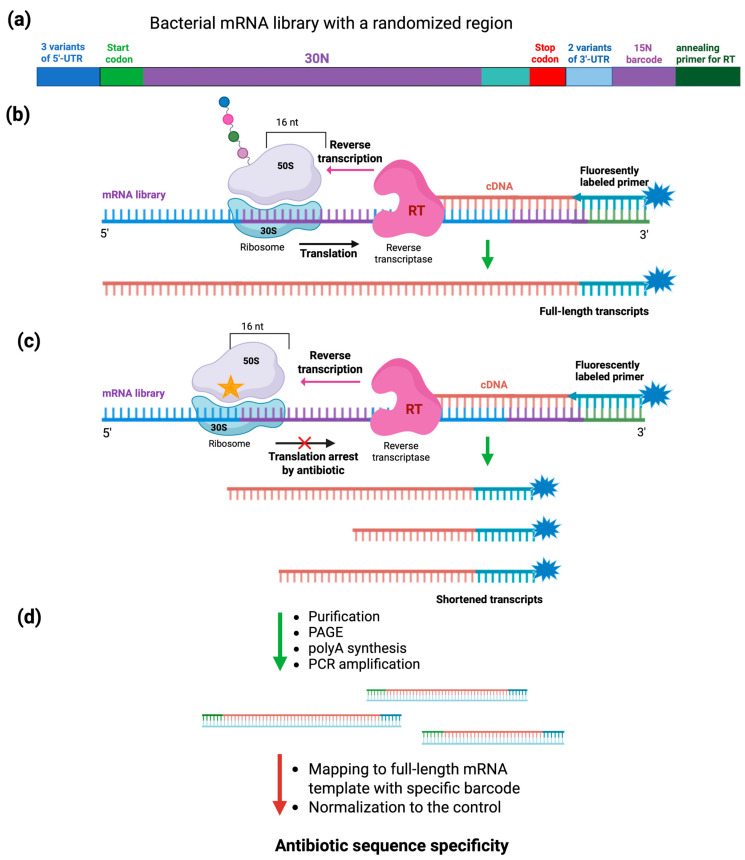
Workflow of Toe-seq. (**a**) Design of mRNA library with randomized region in ORF. (**b**,**c**) Reverse transcription of mRNAs translated in the absence (**b**) or presence (**c**) of an antibiotic (indicated by a star) generates full-length or truncated cDNAs depending on ribosome position. Translation arrest is marked by a red cross on an arrow. (**d**) Following purification, cDNAs are polyadenylated, amplified, and then subjected to NGS. The cDNA length indicates the ribosome stalling position on each mRNA, while the barcode sequence identifies the ORF variant. Mapping reads to the full-length template and normalizing to control read distribution reveals stalling motifs specific to the tested antibiotic. Created in BioRender. Sergiev, P. (2026) https://BioRender.com/3210cfq (accessed on 11 June 2026).

The recently developed Toe-seq method integrates the classical *in vitro* toeprinting assay with next-generation sequencing (Figure 6, Table 1) [54,56,59]. Like traditional toeprinting, this approach detects primer extension inhibition on translating mRNA when a stalled ribosome blocks reverse transcriptase at a characteristic distance of 16 nucleotides from the ribosomal P-site, allowing precise mapping of ribosome arrest sites with codon resolution. Using an mRNA library instead of individual mRNA molecules enables the detection of ribosome stalling motifs across diverse sequences, allowing for the assessment of ribosome pausing and antibiotic context specificity.

The synthetic mRNA library for Toe-seq contains three 5′-UTR and two 3′-UTR variants to offset potential biases arising from the influence of UTRs on translation. The ORF starts with an AUG codon, followed by 10 randomized codons, six constant codons, and a UAA stop codon. To uniquely ascribe each cDNA product to a particular mRNA variant, a 15-nucleotide randomized barcode sequence is placed upstream of the reverse primer annealing site. This design enables the identification of the mRNA by its barcode sequence and determination of ribosome position by cDNA length for each cDNA-derived read [54].

After initiating coupled T7 transcription and translation in the PURExpress system—with or without antibiotics—reverse transcription is performed. The resulting cDNAs are purified and prepared for PCR by adding poly(A) tails using terminal deoxynucleotidyl transferase (TdT). Following PCR amplification with reverse and 3′-oligo(dT)-containing primers, the amplicons are subjected to NGS [54].

Toe-seq monitors ribosome stalling on thousands of synthetic mRNAs *in vitro*, with diversity exceeding that of natural *E. coli* mRNAs by orders of magnitude. The initial library size is optimized to ensure unequivocal mRNA identification and to generate sufficient reads per mRNA for deducing ribosome distribution. The original DNA library is amplified and reused with several antibiotics, allowing for a comparison of ribosome distribution along each particular mRNA across multiple conditions [54].

Toe-seq has been successfully applied to study context-specific ribosome stalling induced by various antibiotics. The suitability of the method was evaluated using well-studied antibiotics, such as edeine A, which prevents ribosome binding to mRNA; THS and TET, which cause ribosome arrest at the start codon; and BOR, which inhibits Thr-tRNA synthetase, leading to increased ribosome stops at threonine codons compared to the water control. Further, context specificity was checked for CHL, ERY, TcmX, and EtaA [54]; BotA2 [56]; and AZI and its derivative Azi-BB [59]. Context-specific inhibition for CHL, ERY, TcmX, and AZI is observed, mainly consistent with previous findings [35,36,39,41,42,43,45,53,68,70]. Notably, Toe-seq revealed, for the first time, EtaA’s stalling specificity, showing a preference for a charged amino acid or stop codon in the A-site, and hydrophobic amino acids in the P- and E-sites [54]. Recent results also demonstrated BotA2′s sequence specificity, inducing ribosome pausing exclusively when the Gly codon enters the A-site, regardless of P- and E-site codons [56], corroborated by Ribo-seq data [57]. Lastly, Toe-seq revealed that Azi-BB2 [59] exhibits reduced sequence specificity compared to AZI for canonical macrolide-sensitive stalling motifs, previously described by other large-scale methods [35,39,41,42,43,68].

Another *in vitro* approach is ribosome display, which is used for high-throughput selection of highly effective protein or peptide binders against virtually any antigen of interest [84]. This method has been applied for over 25 years for the *in vitro* evolution of functional proteins, as well as for diagnostics and therapeutics.

The initial DNA cassette contains six fragments: a T7 promoter, a 5′ stem loop, a translation initiation sequence at the 5′-end, which is either the prokaryotic Shine–Dalgarno (SD) sequence or the eukaryotic Kozak sequence, a peptide gene of interest flanked by a multiple cloning site, a spacer sequence, and a stem loop at the 3′-end [84]. Suitable spacers include gene III of the filamentous phage M13 for prokaryotic systems, and the Cκ (constant region of Igκ chain) or the CH3 domain of human IgM for eukaryotic systems [130,131].

From large DNA libraries, mRNA pools are generated via *in vitro* transcription and used for *in vitro* translation. During translation, noncovalent ternary complexes form between the polypeptide, ribosome, and mRNA, a hallmark of ribosome display technology. The elimination of translational stop codons on the mRNA ensures that the nascent peptide and its encoding mRNA remain bound to the ribosome [84].

Affinity selection is then performed by capturing synthesized polypeptides bound to target antigens immobilized on platforms or magnetic beads. Following selection, ribosomes are disrupted, and the selected mRNAs are recovered, reverse-transcribed, and PCR-amplified for further rounds of selection or analysis. Iterative cycles and additional manipulations enhance the generation of high-affinity binders. Currently, ribosome display is primarily used to obtain potent antibodies from native, immune, or synthetic repertoires [84].

**Figure 7:**
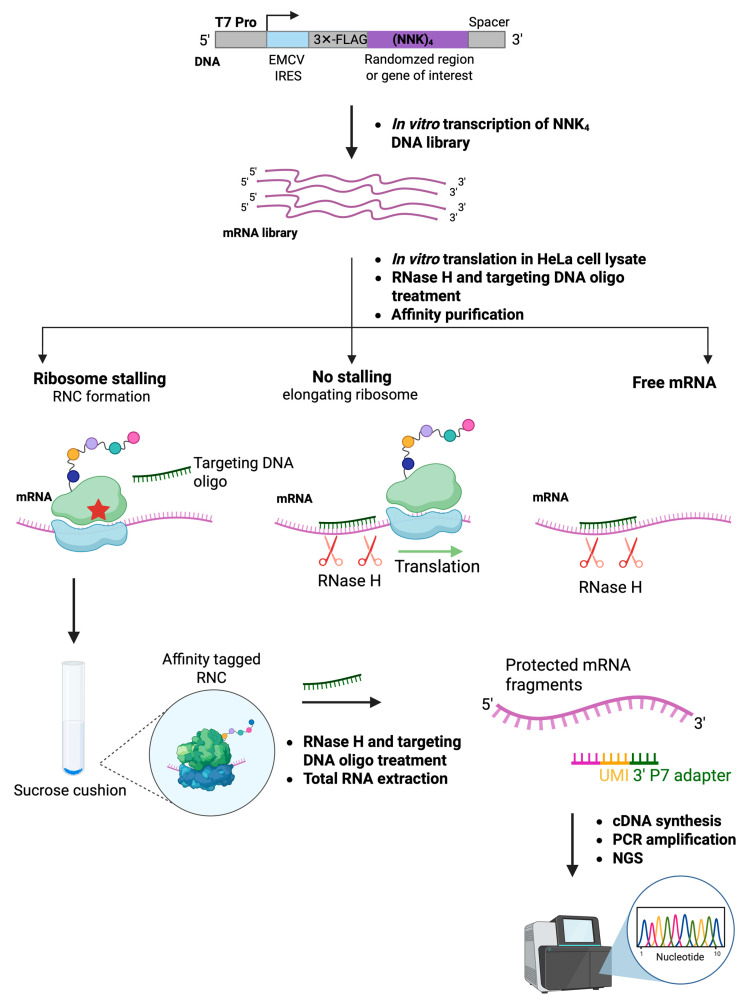
Workflow of ribosome display for selection of stalling sequences from mRNA libraries. The designed mRNA library is translated *in vitro* using HeLa cell lysate in the presence of the tested inhibitor (indicated by a star). The growing peptide is represented as a chain of beads. Selective RNase H cleavage (marked by scissors) removes free mRNAs and transcripts associated with elongating ribosomes, while stalled RNCs are isolated by affinity purification. Extracted total RNA is used for cDNA synthesis with an ORF-specific primer, followed by PCR amplification. Purified PCR products are subjected to deep sequencing to identify ribosome stalling sequences. Created with BioRender. Sergiev, P. (2026) https://BioRender.com/hbd9fsn (accessed on 11 June 2026).

In a modified ribosome display approach (Figure 7, Table 1), the method was used to identify amino acid sequences where human ribosomes stall in the presence of the small molecule PF846 [44]. Previous Ribo-seq studies showed that PF846 selectively stalls ribosomes on a few nascent chains, such as PCSK9, CDH1, and USO1, typically early in their synthesis and without a clear sequence pattern [40].

To gain mechanistic insight, single-particle cryo-EM structures of PF846-stalled ribosome-nascent chain (RNC) complexes were determined. The critical sequences for CDH1 stalling span the nascent chain region near the PTC and extend just beyond the PF846 binding site, where multiple interactions with the ribosome exit tunnel occur. To identify sequence preferences for PF846-mediated stalling, a randomized mRNA library was constructed from CDH1-derived nascent chains. This library was generated by *in vitro* transcription from a T7 promoter and contained an EMCV internal ribosome entry site (IRES), an N-terminal 3X-FLAG tag, and an ORF encoding the CDH1 stalling sequence with stretches of four randomized codons—(NNK)_4_, where N = any nucleotide, K = G or T [44].

This mRNA library was used in an *in vitro* translation system with HeLa cell extract in the presence or absence of PF846. Stalled RNCs were isolated via the FLAG tag at the N terminus of the nascent chain using anti-FLAG beads and were pelleted through a sucrose cushion, retaining mRNAs encoding the stalled nascent chains. To enrich ribosomes stalled at a particular mRNA region, translating complexes were treated with RNase H guided by a DNA oligonucleotide designed to base-pair with the mRNA region protected by a ribosome stalled at the predefined site. This ensured that complexes stalled at the target region were protected from cleavage and preserved for further analysis. The intact mRNAs were then converted into a DNA library and analyzed by deep sequencing. Subsequent analysis of the NGS data enabled the identification of PF846-sensitive stalling sequences [44].

As a result, several PF846-sensitive stalling sequences were identified, strikingly different from the original CDH1 stalling sequence LLLL. Some sequences, such as HYHS and RSCK, preferentially induce PF846-mediated stalling during elongation, while others—NPN*, DPC*, NVI*, and CVT* (where “*” indicates a stop codon)—promote termination arrest by the inhibitor. Further validation using an *in vitro* luciferase assay with mRNAs containing these motifs upstream of the reporter gene of NanoLuc luciferase corroborated these findings [44].

The presented *in vitro* high-throughput approaches allow for the identification of inhibitor-induced ribosome stalling sites in mRNA libraries with randomized coding regions, provided that remaining fragments are unified and other influencing factors are minimized. However, randomization in the coding region inevitably leads to premature stop codons unless the mRNA library has a predefined nucleotide composition. Additionally, multiple stages in preparing ribosome-protected mRNA fragments for NGS can introduce biases in their distribution across the library. To sufficiently increase reads per mRNA for accurate ribosome distribution and stalling analysis, additional strategies are needed, such as optimizing mRNA library size in Toe-seq or performing several rounds of selection in iTP-seq or ribosome display. In iTP-seq, linker ligation can bias mRNA fragment distribution due to nucleotide preferences at fragment ends. In Toe-seq, the use of terminal transferase makes it impossible to distinguish the natural ending nucleotide of a toeprint from the nucleotide added by TdT when they match. Ribosome display is adapted for both bacterial and eukaryotic systems, but faces the added limitation of peptide–ribosome complex instability, which can result in the loss of even stalled complexes. All large-scale methods require intensive computational analysis to identify drug-specific arrest motifs.

### 2.3. Additional Methods for Identifying Ribosome Stalling Sequences

Due to the challenge of predicting novel arrest sequences that lack common motifs, several studies have focused on genome-wide analyses to identify translation-stalling motifs. Methods such as Ribo-seq [35,36,39,48,49,51,55], iTP-seq [43,53,68], Toe-seq [54,56,59], and ribosome display [44] have been used to investigate pause events during translation both in the presence and absence of antibiotics. Additionally, both low- and high-throughput approaches—including *in vivo* and *in vitro* nascent chain profiling (iNP) [86], genetic selection for sequences inducing ribosome stalling [87], and STALL-seq [85]—are employed to identify sequences with ribosome arrest patterns under normal, antibiotic-free conditions. These assays also hold potential for adaptation to detect antibiotic-induced ribosome halting.

**Figure 8:**
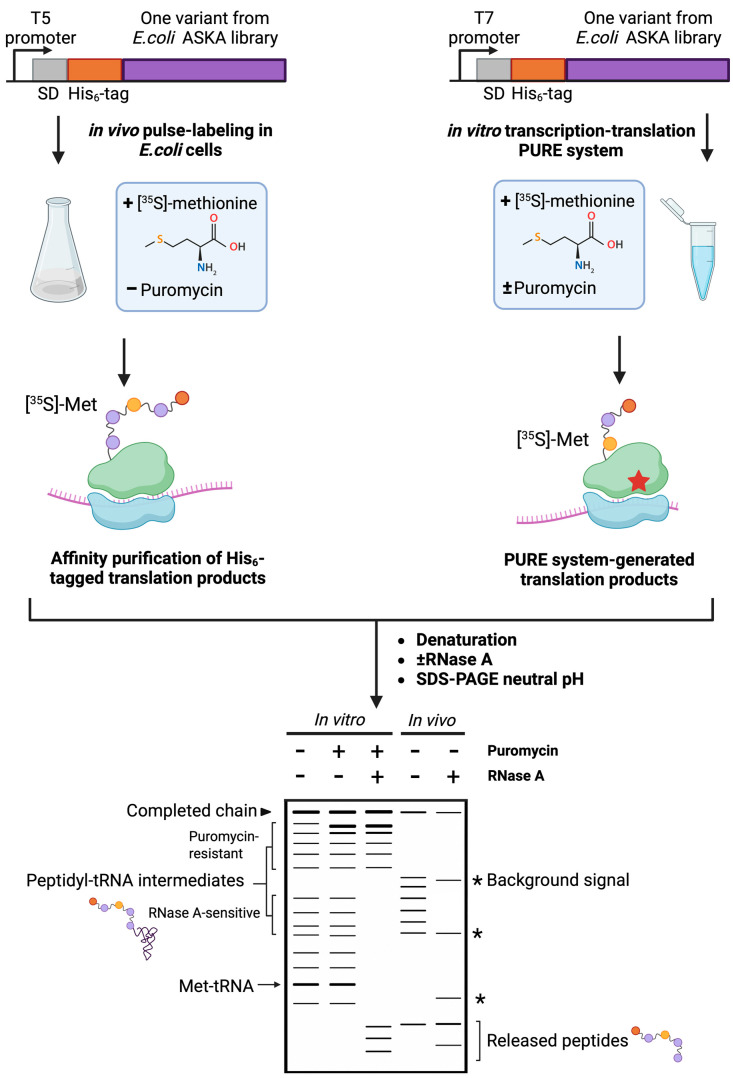
Workflow of integrative profiling of nascent chains *in vivo* and *in vitro* (iNP). The iNP method combines parallel *in vitro* and *in vivo* systems to monitor ribosomal pauses and arrests by detecting nascent polypeptides and peptidyl-tRNAs. Labeled samples are analyzed by SDS-PAGE under neutral pH, with optional puromycin (indicated by a star) and RNase A treatments to distinguish peptidyl-tRNAs from released polypeptides, enabling detection of ribosome stalling and intermediate translation products. The peptide is represented as a chain of beads. Disappearance of a band upon RNase A treatment indicates translational pausing. Puromycin-resistant bands represent *in vitro* pausing intermediates involved in specific mechanisms causing ribosome dysfunction. An arrow indicates the Met-tRNA band, while the asterisks denote background signal bands. Created with BioRender. Sergiev, P. (2026) https://BioRender.com/5ezb6zy (accessed on 11 June 2026).

The low-throughput iNP method combines parallel *in vitro* and *in vivo* systems for monitoring ribosomal pauses and arrests by detecting nascent polypeptides and peptidyl-tRNAs (Figure 8, Table 1) [86]. This approach exploits the fact that peptidyl-tRNAs exhibit slower electrophoretic mobility on neutral pH SDS-PAGE by approximately 18 kDa compared to free polypeptides. This principle enables systematic, individual profiling of nascent chain elongation for 1038 *E. coli* proteins expressed from ASKA library clones, each carrying identical ribosome binding sites and N-terminal coding regions beginning with a 6×His tag [132]. The expression systems utilize the T7 promoter for *in vitro* and the T5-lac promoter for *in vivo* expression [86].

To obtain integrated information on translation elongation, *in vivo* pulse-labeling and *in vitro* PURE system translation were performed in parallel using [^35^S]-methionine for detection. Comparison of *in vitro* and *in vivo* translation elongation allows for the deduction of the role of cellular regulators—absent in the recombinant *in vitro* translation system—in controlling translation elongation [86].

*In vitro* translation reactions are divided into three parts: one is stopped with excess 5% trichloroacetic acid (TCA), and two are optionally treated with puromycin to induce premature peptide release, followed by mixing with 5% TCA and denaturation. One of these puromycin-treated samples is further treated with RNase A to separate peptidyl-tRNAs from released polypeptides [86].

*In vivo*, after cell lysis, radiolabeled translation products are denatured and isolated by His_6_ affinity purification, then split into two parts, with one part additionally treated with RNase A [86].

Samples from both systems are separated by SDS-PAGE under neutral pH. Comparing band migration patterns with or without Puro and RNase A treatment distinguishes peptidyl-tRNAs from free nascent polypeptides, enabling the identification of ribosome stalling sites and intermediate translation products *in vivo* and *in vitro*. Bands disappearing upon RNase A treatment indicate translational pausing. Puromycin sensitivity allows for the assessment of A-site occupancy and/or the ribosome’s ability to perform peptide transfer in the context of stalled complexes [86].

The study identified 2984 translation pause sites *in vitro* across 912 genes and 2541 pause sites *in vivo* across 850 genes, considering each RNase A-sensitive band as a single pausing event. Among these, 969 pauses were *in vitro*-specific (class 1), 526 were *in vivo*-specific (class 2), and 2015 were shared between both systems (class 3), observed in 75% of genes. Notably, genes such as *fliF*, *stfQ*, *araA*, and *secM*, known to contain functionally important pausing sites, as well as genes with stalling polyproline stretches, were identified by the iNP approach, confirming the presence of arrest sequences [86].

Global comparison of data obtained by iNP and Ribo-seq is challenging due to the limited positional resolution of the iNP method and the low expression levels of certain genes in Ribo-seq experiments [133]. Nevertheless, notable correlations were observed for major stalling peaks in *secM*, proline-related pause in *yaaX*, and multisite pauses in *cyoC*. Interestingly, ribosome-profiling peaks attributed to internal SD-like sequences lacked corresponding polypeptidyl-tRNA peaks in iNP data. A plausible explanation is that strong pauses are detected by both approaches, whereas transient or weak pausing—such as local translation slowing caused by internal SD sequences, as suggested by Ribo-seq data in *E. coli* [133]—may not be captured by iNP procedures [86].

**Figure 9:**
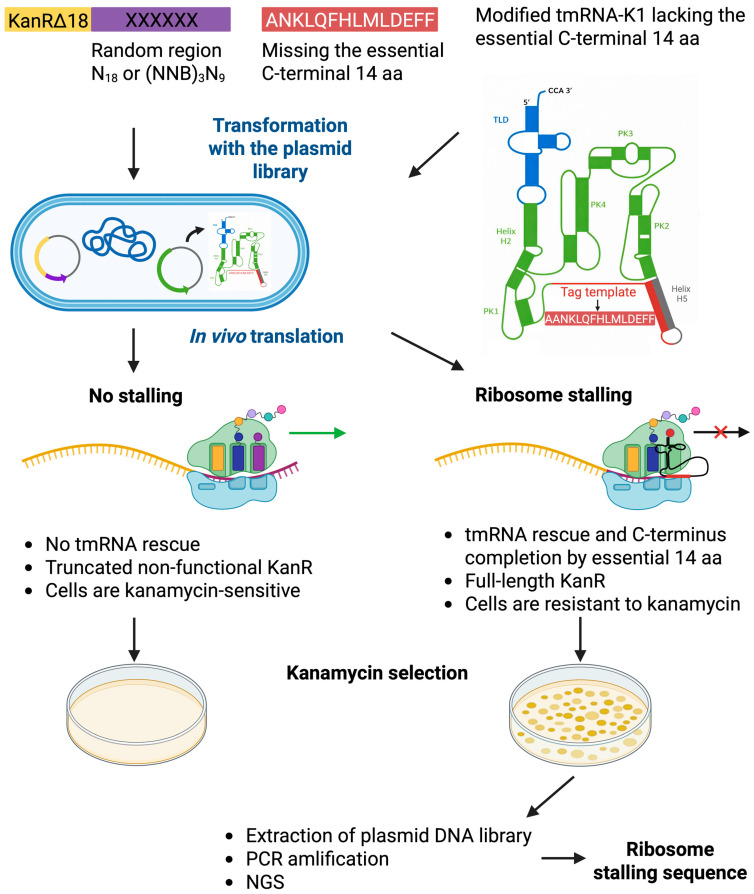
Workflow of genetic selection for nascent peptides that induce ribosome stalling. A library of randomized peptide sequences is fused to a truncated kanamycin resistance protein (KanR∆18), lacking the C-terminal 18 amino acids (highlighted in yellow). If the ribosome translates the mRNA without stalling, no tmRNA rescue occurs, resulting in the synthesis of a truncated, non-functional protein and preventing cell survival on kanamycin plates. In contrast, ribosome stalling triggers rescue by engineered tmRNA-K1, containing a tRNA-like domain (TLD) and restoring the missing C-terminal residues (highlighted in red) and kanamycin resistance. The growing peptide is represented as a chain of beads. Translation arrest is marked by a red cross on an arrow. Sequencing the DNA fragments containing randomized regions from surviving clones allows correlation of ribosome-stalling events with a specific peptide sequence. Created in BioRender. Sergiev, P. (2026) https://BioRender.com/usf00ks (accessed on 11 June 2026).

Another method to identify and characterize translation-stalling peptides is genetic selection, a high-throughput *in vivo* approach that links *E. coli* cell survival to ribosome stalling at specific sites (Figure 9, Table 1) [87]. It exploits the bacterial ribosome rescue system mediated by transfer-messenger RNA (tmRNA), a stable RNA found in eubacteria that tags stalled nascent chains for degradation [134]. tmRNA enters the ribosome’s empty A-site, adds Ala residue to the nascent chain, and serves as a template encoding a short peptide tag recognized by cellular proteases. Once the tag is translated, the ribosome is released at a stop codon within tmRNA, targeting the incomplete protein for destruction.

While tmRNA was initially discovered rescuing ribosomes stalled on mRNAs lacking stop codons [135], it also acts on ribosomes stalled by nascent peptides [136,137]. To utilize this in genetic selection, tmRNA was engineered to complete the synthesis of an essential protein rather than tagging proteins for proteolysis. Specifically, the kanamycin resistance protein (KanR) from Tn10 requires its C-terminal helix (15 amino acids) for activity; truncation of this helix abolishes function. The tmRNA template was modified (tmRNA-K1) to encode the missing C-terminal 14 residue fragment (ANKLQFHLMLDEFF) [138], which, together with the Ala added by aminoacylated tmRNA, restores KanR activity—but only if ribosome stalling occurs precisely at the truncated site [87]. This selection identifies peptide sequences that stall the ribosome at the end of KanR lacking the essential C-terminal helix, thereby recruiting tmRNA, completing KanR synthesis, and conferring kanamycin resistance. Two additional mutations creating a Glu-Pro-(stop) sequence induce stalling during translation termination in truncated KanR; the resulting Glu-Pro-Ala “bridge” formed by stalling and tagging does not impair KanR activity.

To select stalling peptides from random libraries, the truncated *kanR* gene—lacking 18 critical amino acids including the C-terminal helix and three residues in the preceding loop—is fused to a library of randomized six-codon sequences (KanR∆18X_6_). Two randomized region variants are used, N_18_ and (NNB)_3_N_9_, where B presents a mix of G, C and T to limit stop codon occurrence in the fragment. A library of over 10^6^ mutants is generated and introduced along with tmRNA-K1 into an *E. coli* strain lacking wild-type tmRNA. Survival on kanamycin plates depends on sequences that induce ribosome stalling, allowing tmRNA-K1-mediated KanR synthesis completion. Sequencing of survived clones enabled the correlation of ribosome halt with specific peptide motifs. Approximately 1 in 10^4^ colonies survived, indicating that many sequences induce ribosome stalling [87].

This selection identifies not only peptides causing stalling but also nucleic acid sequences eliciting tmRNA tagging, such as rare codon clusters or secondary structures. Analysis of surviving clones revealed three main classes of stalling peptides: peptides with C-terminal proline residues (Pro-stop), SecM-like peptides with consecutive Pro codons (Pro-Pro) at positions three and four without a nearby stop codon, and peptides containing a Trp-Pro-Pro (WPP) motif at positions 1–3 without adjacent stop codons. A novel, highly effective consensus stalling motif—FXXYXIWPP—was identified. Confirmed by toeprinting, immunoblotting, and *lacZ* reporter assays, this motif induces stalling by inhibiting peptidyl transferase activity, like SecM and TnaC leader peptides—retaining peptidyl-tRNA in the P-site and leaving aminoacyl-tRNA unreactive in the A-site. Both the NPET and PTC are involved, though ribosomal mutations affect SecM and FXXYXIWPP arrests differently [87].

Further analysis suggests FXXYXIWPP motif stalls ribosomes via strong aromatic residue interactions within the exit tunnel coupled with inhibition by consecutive proline residues, stabilizing an inactive PTC conformation. These findings reveal that diverse short peptide sequences can cause ribosome stalling, highlighting that translation regulation by nascent polypeptides is more common than previously appreciated [87].

**Figure 10:**
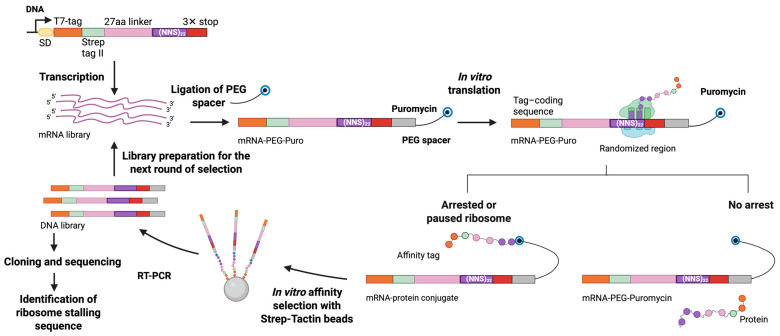
Scheme of the STALL-seq method applied to bacteria. An *in vitro* transcribed mRNA library is enzymatically ligated to a puromycin-PEG spacer (indicated by a line with Puro as a circle at the end) and translated using the PURExpress system. Ribosome pausing or stalling promotes formation of stable mRNA–protein conjugates through puromycin incorporation, whereas completed translation results in peptide and mRNA release. The growing peptide is represented as a chain of beads. The conjugates are enriched by Strep-Tactin affinity purification, followed by RT-PCR amplification. The DNA obtained can be used for subsequent selection rounds or for cloning and sequencing to identify stalling regions. Created in BioRender. Sergiev, P. (2026) https://BioRender.com/7b5e5ok (accessed on 11 June 2026).

A more recent technique for the large-scale investigation of translational arrest peptides acting on both bacterial and eukaryotic ribosomes is STALL-seq (Figure 10, Table 1) [85]. This method combines a modified mRNA display assay [139,140,141] with deep sequencing.

Conventional mRNA display links a cell-free synthesized protein (phenotype) to its encoding mRNA (genotype) by covalently attaching the mRNA to the translated peptide via puromycin, which is attached to the 3′-end of the mRNA without a termination codon through a polyethylene glycol (PEG) spacer. During ribosome stalling at the 3′-end of the mRNA, Puro binds to the ribosome’s A-site, transfers the nascent polypeptide to Puro, and forms stable mRNA–protein conjugates. These conjugates can be selected *in vitro* by affinity to the protein portion [142].

The efficiency of mRNA–protein conjugate formation is drastically reduced by the presence of a termination codon; however, stalling peptides such as SecM enable conjugate formation even with a stop codon present, demonstrating that Puro can bind nascent polypeptides during translational arrest [85]. Thus, both puromycin-sensitive and puromycin-resistant arrest peptides can be selected by mRNA display. Importantly, all these stalling sequences have been identified in *E. coli* proteins using the iNP method [86].

To apply STALL-seq based on mRNA display for the *in vitro* selection of translational arrest sequences, two randomized DNA libraries are constructed. The bacterial library contains a T7 promoter, SD sequence, N-terminal Strep-tag II affinity tag, a 27-amino acid linker, and a 22-codon randomized region (NNS)_22_, where S = G or C (only TAG stop codon can occur), followed by a FLAG tag and triple stop codons in all frames (TAAcTAGcTAA). This library designed to minimize premature stops is translated *in vitro* using the *E. coli* PURE system [142]. The library for translation in a eukaryotic system using wheat germ extract contains an SP6 promoter, the Kozak sequence, the same N-terminal affinity tag, a 50-amino acid linker, a 36-codon randomized region (NNW)_36_, where W = A or T, followed by triple stop codons and a poly(A) tail with puromycin ligated at the 3′-end. *In vitro* translation proceeded normally, releasing peptides when the stop codon entered the ribosome A-site. However, ribosome stalling within the randomized region allows puromycin to bind the nascent polypeptide chain, forming stable mRNA–protein conjugates. These conjugates are recovered using the affinity tag and Strep-tactin beads, followed by RT-PCR and the next selection round, or by cloning and sequencing to identify arrest sequences [85].

STALL-seq revealed the top enriched stalling motifs in both systems. In the bacterial (NNS)_22_ library, sequences with C-terminal polyproline motifs, which are known to cause ribosome halting in the absence of EF-P, were overrepresented. In the eukaryotic (NNW)_36_ library, sequences with Pro-Pro or (K/R)XG(I/V)G motifs at the C-terminus upstream of stop codons were prevalent. The latter motif is novel, with RQGIG being the most frequent variant. Toeprinting assays validated the identified stalling regions [85].

Alongside other described low- and high-throughput *in vivo* and *in vitro* methods, STALL-seq expands the repertoire of techniques for identifying ribosome stalling sequences under normal conditions [85,86,87]. These approaches reveal that ribosome halting is caused by numerous peptide sequences and that it is more common than previously thought. They also hold potential for adaptation to detect antibiotic-induced ribosome arrest.

The additional assays described for identifying ribosome stalling sequences exhibit the typical advantages and limitations of *in vitro* and *in vivo* approaches, as well as low- and high-throughput methods. Among these, only STALL-seq offers the important advantage of being adapted for both bacterial and eukaryotic translation systems. The iNP method provides individual profiling of nascent chain elongation *in vivo* and *in vitro* to monitor ribosome pausing or dysfunction, but it does not reveal ribosome stalling sequences. Both genetic selection and STALL-seq use randomized libraries, which contain premature stop codons, potentially causing false-positive ribosome stalling unless the mRNA library has a predefined nucleotide composition. Furthermore, these large-scale assays require several rounds of selection to distinguish strong ribosome arrest sequences—which may be continuous and depend on exact flanking amino acids—from transient translation pauses.

## 3. Comparative Analysis and Optimization Outlook of Approaches to Study the Context Specificity of Translation Inhibitor Action

### 3.1. Comparative Analysis of Methods for Studying the Context Specificity of Translation Inhibitor Action

The diverse approaches described have broad applications in molecular biology, including identifying sequences with ribosome arrest motifs under normal conditions and studying the context-specific action of translation inhibitors (Table 1). Each method has its advantages and disadvantages, which should be carefully considered when selecting a technology and designing experiments.

*In vivo* assays are performed under native cellular conditions, where natural processes influence translation outcomes. These studies use natural mRNAs of diverse lengths and compositions—as in protein labeling and Ribo-seq—or synthetic constructs based on natural variants, with modifications in specific regions to test single effects within a unified context. Examples include reporter plasmids carrying genes for *β*-galactosidase [43,53,68], luciferase [44] or fluorescent proteins [46,77]; reporter strains with a single rRNA operon (e.g., ChIPS); reporter constructs fused with affinity tags (iNP); and truncated genes like KanR used in genetic selection to detect ribosome pausing and stalling (Table 1). Using different types of mRNAs allows us to examine the sequence region of interest from multiple perspectives and evaluate its effects under various conditions and systems.

The diversity of natural mRNAs *in vivo* is constrained to several thousand forms in bacteria and tens of thousands in eukaryotes, as seen in protein labeling and Ribo-seq. Natural mRNAs likely avoid stalling or pausing sites that are unnecessary for particular regulatory purposes, while their diversity is further limited by uneven expression levels, resulting in overrepresentation of a small subset of highly abundant mRNAs [6]. In contrast, synthetic transcripts enable controlled variability by altering single elements or fragments while maintaining other factors constant. This allows for a focused assessment of specific sequence effects with internal controls and even coverage. However, the diversity of exogenous mRNAs is limited by the design of reporter constructs—single variants in plasmids for complementation assays or in systems like ChIPS, iNP, or plasmid libraries used in genetic selection—and by transformation or transfection efficiencies. Typically, these libraries encompass 10^4^–10^5^ variants [4,64].

The diversity of reporter constructs *in vivo* is generally an order of magnitude greater than that of natural transcripts, whereas the variety of synthetic mRNAs *in vitro* can exceed natural heterogeneity by two orders of magnitude. Therefore, using designed libraries significantly increases the likelihood of encountering ribosome stalling motifs that may be undetectable within natural sequences.

For methods performed entirely *in vitro* (Table 1), two main advantages stand out. First, library diversity is limited only by the number of mRNA variants that are translated by the available ribosomes, translation factors, and energy molecules in the reaction mixture. Second, various lysates and commercially available systems enable targeted *in vitro* translation without interference from other cellular processes, allowing conditions to be tailored flexibly according to research needs. Therefore, it is useful to perform initial screening of ribosome arrest motifs *in vitro*, followed by validation *in vivo* using various approaches of different scales, such as reporter constructs and Ribo-seq.

Among the advantages of low-throughput techniques are the focused testing of a single mRNA at a time and the clear, robust results obtained for each case. These methods are applied both *in vivo*—such as reporter plasmids with *lacZ* or *luc* genes, ChIPS, and iNP—and *in vitro*—such as iNP, toeprinting assays, and cryo-EM. These experiments tend to be reproducible, relatively inexpensive, and fast to perform when the technology is established—except for cryo-EM, which generally requires more time and specific resources. These approaches are currently well suited for verifying ribosome stalling motifs identified during large-scale screenings.

High-performance approaches, on the other hand, are typically multi-stage, costly, and involve longer times with more potential sources of bias. Despite these exceptions, high-throughput methods usually offer broad mRNA diversity and high processivity, allowing for exploration beyond traditional low-throughput techniques (Table 1). These assays often combine ribosomal profiling, toeprinting, inverse toeprinting, or the selection of translation arrest sequences from large libraries coupled with deep sequencing to precisely map ribosome stalling sites and associated sequences. Large-scale technologies are effective for *de novo* discovery of unknown ribosome arrest sequences but require validation of the characterized motifs.

Synthetic transcripts with randomized ORF regions encompass many more variants and potential ribosome stalling patterns—often up to one hundred thousand unique mRNA sequences per variable fragment—far surpassing the diversity achievable *in vivo* and free from intracellular biases. Two key considerations arise in such studies. First, the third nucleotide of each codon is often altered to limit stop codon emergence and focus on amino acid motifs. Common variations include (NNB)_n_ [87], (NNK)_n_ [44], (NNS)_n_ [43,68,85], and (NNW)_n_ [85], rather than the full (NNN)_n_ diversity [53,54,56,59,87]. Additionally, not all release factors may be present in the translation reactions [43,53,68]. Designing an mRNA library with a preset composition seems to be a reasonable solution to this problem. Second, the library size must be constrained to reliably assess stalling efficiency on specific sequences, ensuring substantial coverage of individual mRNA variants. Without limiting diversity, each sequenced read could originate from a different mRNA species, making it impossible to determine ribosome distribution along a particular mRNA. Solutions include adjusting template molecule numbers so ribosomes repeatedly stall on specific transcript variants, as demonstrated by Toe-seq data [54,56,59], or performing multiple selection rounds of ribosome-protected mRNA fragments, as implemented in the initial iTP-seq application [43].

Furthermore, the ribosome display method facilitates the introduction of random mutations after each selection round without the need to re-transform cells with the library, thereby accelerating the directed evolution of binding proteins over multiple generations [143].

Among large-scale assays, those employing sequence-specific enzymes—such as nucleases and RNA ligases used in Ribo-seq, iTP-seq, ribosome display, and STALL-seq—require additional data processing to correct for enzyme preferences [118,125,126,127]. Ideally, supplementary experiments should be conducted to minimize biases introduced by these enzymes. The Toe-seq method employs terminal transferase to add a poly(A) tail to cDNA molecules, thus creating a challenge in distinguishing terminal adenosines added during reverse transcription from those appended by TdT [54,56,59]. To reduce this bias, using alternative tails like poly(C) can help, provided that cytidine is avoided in the constant coding region. However, poly(G) or poly(U) tails are less preferable since they complicate distinguishing from possible terminal nucleotides in start or stop codons. Additionally, performing as least two parallel experiments on the same samples—dividing them to treat with different nucleases (as in Ribo-seq) or preparing libraries by linker ligation and TdT-tailing (common to all four approaches)—is advisable to improve reliability. Furthermore, large-scale sequencing data demand advanced computational skills for processing, as manual analysis of all sequences is impractical. Given the multiple stages in high-throughput research where reliability issues may arise, results should be validated using low-throughput methods such as reporter constructs (to check *in vivo* effects), toeprinting, and/or cryo-EM (to confirm *in vitro* ribosome stalling at nucleotide resolution) (Table 1).

### 3.2. Comparative Analysis of Mainstream High-Throughput Methods for Studying Context Specificity of Translation Inhibitor Action

When comparing three mainstream high-throughput platforms—Ribo-seq, iTP-seq, and Toe-seq—Ribo-seq identifies antibiotic-induced ribosome stalling patterns in diverse natural mRNA libraries during protein biosynthesis in living bacterial and eukaryotic cells. In contrast, iTP-seq and Toe-seq mostly analyze randomized coding regions during *in vitro* translation in purified bacterial systems. Challenges with Ribo-seq primarily occur during ribosome-protected footprint generation using sequence-specific enzymes and library preparation for amplification and NGS. iTP-seq shares these issues, while for Toe-seq, only library preparation is problematic.

The most accurate *de novo* identification of unknown inhibitor-induced ribosome stalling motifs occurs when consistent sequences are obtained by all three techniques or at least by Ribo-seq *in vivo* and either iTP-seq or Toe-seq *in vitro*. Their applicable workflows are as follows. Ribo-seq: polysome extraction from frozen cells, footprint generation by different enzymes in parallel, PAGE size selection of footprints, specific sequence addition via linker ligation or TdT-tailing, and deep sequencing. iTP-seq: inverse toeprint generation by RNase R, specific sequence addition by two methods, PAGE size selection, and NGS. Toe-seq: toeprint generation by RT, specific sequence addition by two methods, and NGS. Toe-seq involves fewer steps, reducing time and bias.

Designing a synthetic mRNA library with a preset composition that excludes stop codons and enriches specific amino acid and codon combinations is a reasonable strategy to maximize sequence diversity when screening ribosome stalling patterns induced by translation inhibitors, while addressing the inherent challenges of such libraries.

Additionally, ribosome profiling can be adapted to *in vitro* conditions using cell lysates with natural or synthetic mRNA libraries, allowing comparisons with *in vivo* Ribo-seq and *in vitro* iTP-seq or Toe-seq results for cross-verification of ribosome arrest patterns.

### 3.3. Optimization Outlook of Additional Methods for Identifying Ribosome Stalling Sequences

Some large-scale assays, such as iNP [86], genetic selection for sequences inducing ribosome stops [87], and STALL-seq [85] (Table 1), have not yet been applied to investigate antibiotic sequence specificity. However, these methods have identified both common and novel ribosome arrest motifs and hold promise for adaptation to define the context specificity of translation inhibitors.

This adaptation can be achieved by introducing antibiotic selective pressure into screening systems. However, inhibitors with binding sites identical or close to those of puromycin, chloramphenicol, or kanamycin should be avoided, as these antibiotics are used for sample treatment in iNP, genetic selection, and STALL-seq, respectively. Additionally, designing a synthetic mRNA library with a preset composition is recommended for high-throughput genetic selection and STALL-seq techniques. For randomized library preparation in STALL-seq, both TdT-tailing and linker ligation should be employed to minimize potential biases.

### 3.4. Multi-Technique Combined Validation Strategy for Studying Context Specificity of Translation Inhibitor Action

For several translation inhibitors, consistent results have been obtained using the various techniques described. The reproducibility of stalling motifs across multiple assays validates both the findings and the methods.

Stalling motifs can be quite stringent, as seen with CHL, LZD, and BotA2. Notably, CHL’s sequence preference shows strong convergence across *in vivo* and *in vitro*, low- and high-throughput methods, demonstrating pronounced specificity for Ala, and—to a lesser extent—Ser, Gly, and Thr codons in the ribosome’s E-site [36,45,54,55,70].

Conversely, arresting motifs can be diverse and governed by sets of preference rules, especially for NPET inhibitors acting as allosteric modulators. These inhibitors cause ribosome halting at variable combinations of polymerized amino acids that cannot be easily summarized by a single motif. This situation is observed with macrolides (ERY, AZI), ketolides (TEL, SOL), and TcmX and EtaA, all of which display variable stalling patterns. Such cases highlight the importance of employing multiple approaches for the cross-verification of sequence specificities. Understanding the mechanisms of translation inhibitor action—including their sequence specificity—could accelerate the development of less toxic, highly specific antimicrobial agents that selectively suppress the synthesis of a limited set of proteins. Advanced context-specific inhibitors targeting the PTC and NPET, known as interdictors, preferentially interact with nascent protein residues exhibiting complementary physiochemical properties to small-molecule moieties. This interaction induces structural rearrangements in both the nascent polypeptide chain and ribosomal RNA. Additionally, these compounds differentially modulate ribosome surveillance pathways, including the ribotoxic stress response. Their anti-tumor activity has also been investigated through binding to human ribosomes and stalling translation of disease-driving oncoproteins [144]. Although the design of such molecules remains rare, it represents an urgent and promising area of research.

To characterize known or newly discovered stalling motifs for a translation inhibitor in detail, the following research pipeline with optimal technical combinations is recommended. First of all, protein labeling should be performed *in vivo* and/or *in vitro* in the presence of the antibiotic of interest to detect selective protein synthesis. Classic toeprinting with several mRNAs is also useful for the initial detection of ribosome stalling caused by the drug. Secondly, if selective protein synthesis or context specificity is observed, a high-throughput *in vivo* or *in vitro* approach such as Ribo-seq, iTP-seq, or Toe-seq should be applied. Thirdly, upon identifying stalling motifs, strong ribosome arrest sequences should be validated using low-throughput assays like *in vitro* toeprinting and/or *in vivo* reporter constructs. Then, the structural resolution of ribosome–inhibitor complexes should be obtained from the bacterial system. After that, additional large-scale mainstream methods should be conducted to confirm the identified arrest motifs, especially for antibiotics binding in the ribosome NPET that demonstrate diverse stalling patterns. Last, high-performance techniques should be performed in eukaryotic systems, such as ribosome display, combined with structural studies on human and mitoribosomes to provide a comprehensive analysis of the inhibitor. This multi-faceted approach ensures robust and reliable characterization of context-specific antibiotic-induced ribosome halting during translation.

## 4. Conclusions

Previously, low-throughput approaches using single mRNA, such as reporter constructs, ChIPS, toeprinting, and cryo-EM, served as the primary sources for studying the context specificity of translation inhibitors or provided initial insights. Protein labeling first offered high-throughput information on selective protein synthesis in the presence of macrolide antibiotics.

With the advent of modern high-performance methods analyzing mRNA libraries, such as Ribo-seq, iTP-seq, Toe-seq, and ribosome display (Table 1), these earlier low-throughput assays are now mainly used to validate findings from large-scale studies. Among these, Ribo-seq, iTP-seq, and Toe-seq have become the primary mainstream assays for identifying context-specific antibiotic-induced ribosome stalling. However, their limitations—related to sequence-specific enzymes and library preparation—necessitate the use of diverse parallel experimental procedures or different high-throughput approaches to minimize biases and confirm the observed stalling motifs. *In vitro* methods also require synthetic mRNA libraries with preset compositions to maximize sequence diversity for screening inhibitor-induced ribosome stalling patterns.

Nevertheless, all methodologies remain essential for confirming results on the sequence specificity of translation inhibitors, whether *in vivo* or *in vitro*, and whether low- or high-throughput, considering each approach’s advantages, limitations, and challenges. Most approaches form the foundation of research pipelines that ensure accurate, detailed characterization of sequence-specific inhibitor-induced ribosome stalling during protein biosynthesis. This array of techniques is complemented by methods such as iNP, genetic selection, and STALL-seq (Table 1), which hold potential for studying context-specific antibiotic action when they are appropriately modified by introducing antibiotic selective pressure into screening systems.

The nature of the inhibitor and its ribosome binding site strongly influence its spectrum of stalling patterns. These can follow quite stringent rules, as seen with CHL, LZD, and BotA2, or can be diverse and governed by preference sets, especially for NPET inhibitors acting as allosteric modulators, such as macrolides, ketolides, TcmX, and EtaA. Such observations underscore the importance of employing multiple approaches for cross-verification of sequence specificities, organized within a comprehensive experimental pipeline.

The fundamental scientific value of this review lies in establishing standardized experimental selection guidelines for researchers investigating ribosome stalling mechanisms, thereby minimizing trial-and-error costs. Equally important is its applied relevance: understanding the mechanisms underlying protein biosynthesis inhibition and their dependence on specific mRNA sequences can enable the development of selective agents that precisely suppress the synthesis of targeted polypeptides, such as proteins from pathogenic bacteria or cancer-associated proteins.

This review provides a detailed overview of diverse approaches used to identify ribosome stalling motifs and verify the sequence specificity of various translation inhibitors. It systematically classifies all low- and high-throughput, *in vivo* and *in vitro* assays and offers a comparative analysis of each technique, highlighting their advantages, limitations, compatible biological systems, and representatives of different inhibitor categories for which they were used. Furthermore, it proposes experimental selection strategies and their optimization outlook, as well as combined screening and validation pipelines that integrate low- and high-throughput platforms to guide future mechanistic studies of translation inhibitors and highlight trends in their specific development.

## Figures and Tables

**Table 1:** Various methods for analyzing the context specificity of translation inhibitors.

Method	*In Vivo*/ *In Vitro*	Low- or High-Throughput Method	mRNA Type	Application to Bacteria and/or Eukaryotes	Inhibitor Selectivity Testing	Sequence- Specific Enzyme Usage	References
Reporter constructions with *lacZ* or *luc* reporter gene	*in vivo*	Low-throughput	Biosynthesized from single type plasmid with single motif	Bacterial/ Eukaryotic	ERY, AZI, CHL, TEL	No	[28]
					ERY, Ole		[43]
					ERY, TEL		[68]
					TcmX		[53]
					PF846		[44]
					MKM		[58]
Dual-fluorescent protein reporters	*in vivo*	High-throughput	Biosynthesized from single type plasmid with single motif	Bacterial	TcmX	No	[46]
					ERY, AZI, CHL, EtaA		[77]
ChIPS	*in vivo*, *in vitro*	Low-throughput	Single rRNA operon	Bacterial	PAC, CHL	No	[34]
Toeprinting assay	*in vitro*	Low-throughput	Synthetic single mRNA	Bacterial/ Eukaryotic	HT, homoharringtonine	No	[32,38]
					ERY, TEL		[68]
					TcmX		[46,53]
					ERY, TEL, CHL, LZD, EVN		[34]
					ERY, TcmX, EtaA, THS, TET, BOR		[54]
					THR, RET		[52]
					AZI		[35,59]
					BotA2		[56,57]
					MKM, RET		[58]
Cryo-EM	*in vitro*	Low-throughput	Synthetic single mRNA	Bacterial/ Eukaryotic	ERY	No	[46,71,72,99]
					ERY, TEL		[42,48,68]
					TcmX		[46,53]
					EtaA		[54]
					LZD, RZD		[50]
					CHL		[70]
					CHL, LZD		[55]
					MKM		[58]
Protein labeling	*in vivo*/ *in vitro*	High-throughput	Natural cellular or synthetic mRNAs	Bacterial/ Eukaryotic	ERY	No	[100]
					SOL		[48]
					ERY, TEL, PKM		[35,78]
					MTM, PKM		[37]
Ribo-seq	*in vivo*	High-throughput	Natural cellular mRNAs	Bacterial/ Eukaryotic	ERY, TEL	Yes (RNase I, MNase, T4 RNA ligase truncated)	[39]
					TEL		[48]
					AZI		[35]
					CHL, LZD		[36,55]
					EVN		[51]
					KSG		[49]
					RET		[83]
					MKM		[58]
iTP-seq	*in vitro*	High-throughput	Synthetic randomized libraries (NNS)_15_ or (NNN)_15_	Bacterial	ERY, Ole	Yes (RNase R, T4 RNA ligase truncated, restrictases)	[43]
					ERY, TEL		[68]
					TcmX		[53]
Toe-seq	*in vitro*	High-throughput	Synthetic mRNA library (10 randomized codons)	Bacterial	CHL, ERY, THS, TET, TcmX, EtaA, BOR	No	[54]
					BotA2		[56]
					AZI, Azi-BB		[59]
Ribosome display	*in vitro*	High-throughput	Synthetic mRNA library (4 randomized codons NNK_4_)	Bacterial/ Eukaryotic	PF-06446846 (PF846)	Yes (RNase H)	[44]
iNP (*in vivo*/*in vitro* nascent chain profiling)	*in vitro*, *in vivo*	Low-throughput	Biosynthesized from single type plasmid and single synthetic mRNA	Bacterial	CHL (for *in vivo* selection only) Puromycin (Puro, for *in vitro* selection only)	Yes (RNase A)	[86]
Genetic selection	*in vivo*	High-throughput	Synthetic mRNA library (6 randomized codons N_18_ or (NNB)_3_N_9_) of the modified natural mRNA	Bacterial	Kanamycin (Kan, for *in vivo* selection only)	No	[87]
STALL-seq	*in vitro*	High-throughput	Synthetic mRNA library (NNS_22_, NNW_36_)	Bacterial/ Eukaryotic	Puro (for *in vitro* mRNA–protein conjugate generation only)	Yes (T4 RNA ligase)	[85]

## Data Availability

No new data were created or analyzed in this study. Data sharing is not applicable to this article.

## Abbreviations

The following abbreviations are used in this manuscript:

Inhibitors	
ERY	erythromycin
AZI	azithromycin
TEL	telithromycin
SOL	solithromycin
HT	harringtonine
PAC	pactamycin
CHL	chloramphenicol
LZD	linezolid
MTM	methymycin
PKM	pikromycin
PF846	small-molecule inhibitor PF-06446846
Ole	oleandomycin
KSG	kasugamycin
TcmX	tetracenomycin X
RZD	radezolid
EVN	evernimicin
THR	thermorubin
EtaA	etamycin A
BotA2	bottromycin A2
MKM	manikomycin
Azi-BB	azithromycin benzoxaborole derivative
MLS	macrolide–lincosamide–streptogramin B group antibiotics
BOR	borrelidin
TET	tetracycline
THS	thiostrepton
RET	retapamulin
Puro	puromycin
Kan	kanamycin
Others	
mRNA	messenger ribonucleic acid
tRNA	transfer RNA
rRNA	ribosomal RNA
UTR	untranslated region
ORF	open reading frame
NPET	nascent peptide exit tunnel
2D, 3D	two-, three-dimensional
RT	reverse transcription
PTC	peptidyl transferase center
RiPP	ribosomally synthesized and post-translationally modified peptide
cryo-EM	cryogenic electron microscopy
ChIPS	characterization of inhibitors of protein synthesis
NGS	next-generation sequencing
Ribo-seq	ribosome profiling with following NGS
iTP-seq	inverse toeprinting coupled with NGS
Toe-seq	high-throughput toeprinting and next-generation sequencing
STALL-seq	selection of translational arrest sequences from large library sequencing
iNP	in vivo and in vitro nascent chain profiling
*E. coli*	*Escherichia coli*
Erm	erythromycin resistance methyltransferase
RBS	ribosome binding site
*lacZ*	*β*-galactosidase gene
cDNA	complementary DNA
nt	nucleotides
SRC	stalled ribosome complex
MAM	macrolide-arrest motif
uORF	upstream open reading frame
[^35^S]-Met	methionine labeled with the radioactive isotope [^35^S]
[^14^C]-Val	valine labeled with the radioactive isotope [^14^C]
HPG	homopropargylglycine
OPP	O-propargyl-puromycin
PAGE	polyacrylamide gel electrophoresis
RNase	ribonuclease
MNase	micrococcal nuclease
PCR	polymerase chain reaction
poly(A)	polyadenylated sequence
oligo-d(T)	oligonucleotide of repeating deoxythymidine residues
ss	single-stranded
RF1, RF2, RF3	release factors 1, 2, 3
dsDNA	double-stranded DNA
TdT	terminal deoxynucleotidyl transferase
SD	Shine–Dalgarno sequence
RNC	ribosome-nascent chain complex
IRES	internal ribosome entry site
RPF	ribosome-protected fragment
TCA	trichloroacetic acid
tmRNA	transfer-messenger RNA
KanR	kanamycin resistance protein
PEG	polyethylene glycol
